# Surgical Treatment of “Large Uterine Masses” in Pregnancy: A Single-Center Experience

**DOI:** 10.3390/ijerph182212139

**Published:** 2021-11-19

**Authors:** Anna Franca Cavaliere, Annalisa Vidiri, Salvatore Gueli Alletti, Anna Fagotti, Maria Concetta La Milia, Silvia Perossini, Stefano Restaino, Giuseppe Vizzielli, Antonio Lanzone, Giovanni Scambia

**Affiliations:** 1 Obstetrics and Gynaecology Unit, Santo Stefano Hospital, AUSL Toscana Centro, 59100 Prato, Italy; 2 Università Cattolica del Sacro Cuore, 00168 Rome, Italy; annalisavidiri@gmail.com (A.V.); conci.lamilia@gmail.com (M.C.L.M.); silvia.perossini@gmail.com (S.P.); antonio.lanzone@policlinicogemelli.it (A.L.); giovanni.scambia@policlinicogemelli.it (G.S.); 3 Division of Gynecologic Oncology, Fondazione Policlinico Universitario A. Gemelli IRCCS, 00168 Rome, Italy; salvatore.guelialletti@policlinicogemelli.it (S.G.A.); anna.fagotti@policlinicogemelli.it (A.F.); 4 Division of Obstetrics and Gynecology, University Hospital of Udine, Azienda Sanitaria Universitaria Friuli Centrale, 33100 Udine, Italy; stefano.restaino@asufc.sanita.fvg.it (S.R.); giuseppe.vizzielli@asufc.sanita.fvg.it (G.V.); 5 Department of Woman and Child Health and Public Health, Fondazione Policlinico Universitario A. Gemelli IRCCS, 00168 Rome, Italy

**Keywords:** myomectomy during pregnancy, large mass, obstetric complications, uterine myomas

## Abstract

Uterine myomas or uterine fibroids are the most common benign uterine masses affecting women. The management of large myoma during pregnancy is challenging, and surgical treatment is a possible option. We report nine cases of pregnant women affected by uterine masses larger than 10 cm, who underwent surgical treatment during the second trimester of pregnancy. In all cases, the masses were preconceptionally unknown and diagnosed during the first trimester. In eight cases, no maternal and fetal complications arose during or after surgical treatment and delivery occurred at full term of pregnancy. In one case, spontaneous abortion was recorded. In all cases, histologic diagnosis demonstrated the benign nature. Women affected by large uterine masses diagnosed for the first time in pregnancy could be taken into consideration for surgical treatment in a referral center during the second trimester.

## 1. Introduction

Uterine myomas or uterine fibroids are the most common benign uterine neoplasms, starting at reproductive age [1,2,3]. In obstetric clinical practice, it is possible to face uterine myomas, but they cannot be easily assessed and the prevalence ranges between 0.1 and 3.9% [4].

Uterine fibroids are symptomatic in about 25% of cases [2,3], and in pregnancy they have been associated with increased risks of spontaneous abortion, preterm delivery, preterm premature rupture of membranes (pPROM), bleeding during pregnancy and postpartum hemorrhage, placental abruption, fetal malpresentation, dystocia and cesarean delivery [5].

Although the management of myomas in non-pregnant women follows clear guidelines outlining different options, which range from medical to surgical treatment [6], not all such treatments are suitable for pregnant women due to the possible risk of fetal and placental damage. Therefore, if conservative management is the preferred therapeutic choice, myomectomy is generally a feasible option in cases such as torsion of pedunculated fibroids, necrosis, severe maternal symptoms or large/rapidly growing myomas located in the lower segment of the uterus deforming the placental site [7].

Diagnosis and follow up of uterine masses are mainly based on ultrasound evaluation [8], and the possibility of leiomyosarcoma should be called into question. When the diagnosis of a large pelvic mass is made during pregnancy, even if compatible with myoma, differential diagnosis should consider malignancy, such as ovarian cancer or seldom leiomyosarcoma [9,10].

We report a case series of pregnant women with large uterine mass(es), which were unknown in the preconceptional period and revealed for the first time in early pregnancy, who underwent surgical treatment during the second trimester of gestation.

## 2. Materials and Methods

Nine pregnant women with large uterine mass(es) diagnosed for the first time in pregnancy between September 2016 and January 2020 were retrospectively recorded. The inclusion criteria were as follows: an ultrasound diagnosis performed for the first time in pregnancy of at least one mass 10 cm in size or bigger, which we classified as “large uterine mass”; and first spontaneous pregnancy. The exclusion criteria were as follows: patients who denied consent for surgical treatment, previous abdominal surgery, thickness of residual myometrium between the mass and the uterine cavity ≤5 mm and women with uterine mass known for a long time before pregnancy (<10 cm).

To reduce the risk of chorionic membrane trauma during surgical resection, ultrasound evaluation included detailed mapping of the mass (in particular, the site of attachment), assessment of the pedicle (if present) and myometrial thickness. Ultrasound scans (US) were performed transvaginally (GE RIC5-9H 3D-4D Transvaginal Probe) and transabdominally (GE C1-5-D, Convex Probe) by a single physician with specific training in obstetric sonography, using a Voluson E8 system (GE Healthcare Zipf, Tiefenbach, Austria). In all cases, diagnosis was performed during the first trimester, between 9 and 13 weeks of gestational age.

For each case, a detailed briefing was performed by an experienced team of gynecologists to evaluate the risks and benefits of surgical treatment, considering mass(es) volume and site, the unknown nature and the missing evidence of their presence before pregnancy. All patients gave consent to undergo the procedure, which was scheduled during the second trimester of pregnancy, when the risks of spontaneous abortion and preterm contractions are lower, making it the optimal time for elective surgical treatment. Patients were extensively counseled on (a) increased risks of poor pregnancy outcome, especially premature birth, (b) increased risk of maternal organ compression due to mass(es) dimensions and (c) the need of a conclusive histological diagnosis to rule out malignancies (sarcoma).

**Figure 1:**
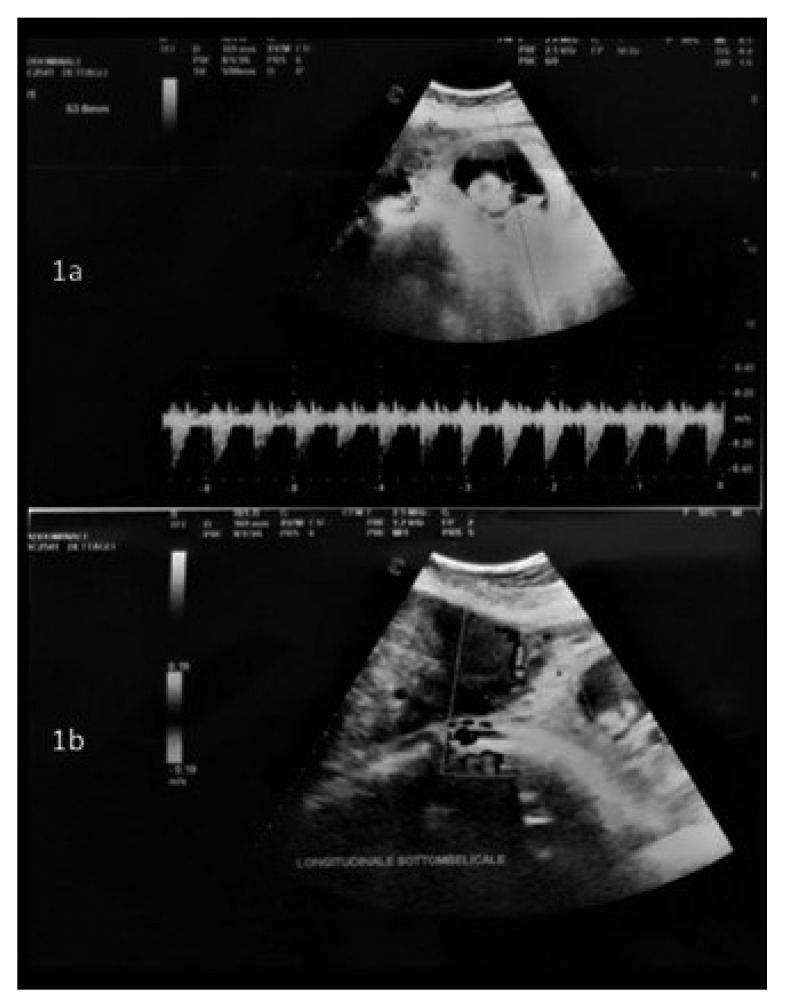
(**a**) Doppler ultrasound to check fetal heartbeat; (**b**) Assessment of the position of larger myoma and its pedicle.

**Figure 2:**
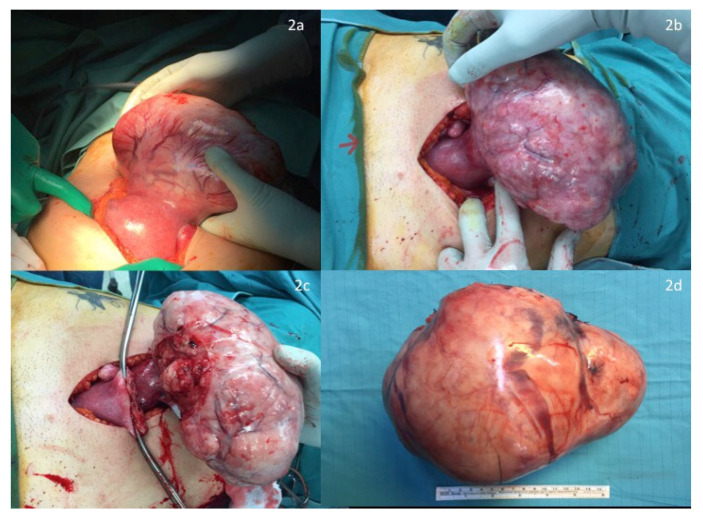
(**a**,**b**) Exteriorization of large mass after longitudinal laparotomy. (**c**,**d**) Surgical resection along the surface.

Before surgery, an intraoperative ultrasound was performed to assess both the fetal heart rate (Figure 1a) and the position of the larger mass and its pedicle (Figure 1b). Surgery was performed in all cases under general anesthesia, using a longitudinal incision to achieve the best control of the surgical field; this determined the maximum surgical efficacy with as minimal as possible manipulation of the uterus. A careful exploration of the surgical field, defining the number, the site and the position of all masses, enabled the surgeon to further confirm the surgical strategy. Exteriorization of the large mass was subsequently performed to clarify the dimensions of its pedicle and to start the surgical resection along its surface to save enough tissue to perform hysterorrhaphy, thus reducing the stitching on the “functional myometrium” (Figure 2a–d). In case of a large pedicle, the simultaneous forcipressure of the resected tissue ensured good control of the hemostasis, reducing the number of required stitches (Figure 2c). At the end of the surgery, fetal vitality was checked and confirmed by the presence of normal fetal cardiac activity, regular amniotic fluid and normal placenta adhesion. 

Pharmacologic therapy with antibiotics and progesterone was administered. All patients underwent a monthly follow-up until their delivery. 

## 3. Results

From September 2016 to January 2020, nine pregnant women with large myomas were surgically treated during the second trimester of pregnancy, and at least one large uterine mass, >10 cm, was removed. Patients’ characteristics are reported in Table 1. In seven cases, no severe symptoms were reported despite the mass occupying the pelvis and abdomen up to the subdiaphragmatic site. Pelvic pain and vaginal bleeding were reported in the remaining cases. 

Large masses were all subserous and in six cases they were localized in the right part of the uterine fundus, extending up to the lower surface of the liver. US characteristics of masses are reported in Table 2.

Out of three patients, only one mass was excised, while in the remaining six cases, two or more masses were removed (Table 2). Intraoperative blood loss ranged between 200 cc and 1400 cc, but blood transfusion was only necessary in one case (Table 2) for severe anemia. The mean duration of the surgical intervention was 105 min (ranging from 90 to 135 min). Postoperative hospitalization ranged from 4 to 7 days. The histologic diagnosis demonstrated the benign nature of all 27 excised masses. Regarding the obstetric outcome, in all but one case the pregnancies were carried to full term without maternal or fetal complications. As for the only adverse outcome, 8 h after surgical treatment with excision of multiple myomas, of which two were larger than 10 cm and four were larger than 5 cm, spontaneous abortion occurred with spontaneous fetal expulsion. The blood loss was substantial (1400 cc) and required perioperative transfusions. No hysterectomy was needed. 

In the other eight successful cases, women were closely followed up with ultrasound and physical examination every four weeks until delivery, and a cesarean section was performed after the 37th gestational week. More specifically, in seven cases the delivery was through an elective cesarean section, while one was a scheduled cesarean section due to premature rupture of membranes (PROM). No complications related to the cesarean sections and the feto-neonatal outcomes were reported.

## 4. Discussion

The relationship between fibroids and pregnancy outcome is not supported by high-quality data. However, the available information, mainly from observational case series, reports that the presence of myoma(s) during pregnancy is associated with an increased risk of maternal, fetal and neonatal complications [5], and the risk of pregnancy loss or preterm delivery may be higher when there are multiple fibroids [5,11]. 

The appropriate therapeutic approach of myomas/masses during pregnancy depends on several factors, considering the severity of symptoms, size, number and site of masses. Moreover, if a large (>10 cm) uterine mass is diagnosed for the first time in pregnancy, there are no ultrasonographic or MRI features to decisively differentiate a benign lesion (uterine leiomyoma) from a malignant variant (uterine sarcoma) [12]. 

To the best of our knowledge, two main approaches for the management of myomas during pregnancy have been described: (i) conservative treatment with observation and, when required, analgesic and tocolytic treatments [13]; and (ii) surgical treatment, mainly after the failure of conservative approaches and/or when a rapid abnormal growth and increase in size with displacement of the surrounding organs is observed. 

The data in the literature describe both laparoscopic and laparotomic surgical procedures. However, in cases with large masses, the laparotomic approach gives the advantage of achieving the best control of the surgical field and extracting the myoma intact, especially when the malignant nature cannot be ruled out. Indeed, in 2014, the Food and Drug Administration released a statement advising against the use of laparoscopic morcellation in cases with large myomas of unknown origin [12,14,15,16].

Nevertheless, in our research we decided to surgically treat the asymptomatic patient as well, which was first performed during pregnancy, due to the large size at diagnosis, the uncertain nature of the detected mass and the potential pregnancy complications. In the cases considered here, we removed small myomas only when they were subserous or pedunculated, and strictly close to the large myoma. Although a surgical approach for uterine myomas during pregnancy is not the preferred choice, our experience suggests that it can be performed, in selected cases, by an experienced team of surgeons after an accurate preoperative multidisciplinary planning.

In our study, we selected cases where the women presented large pelvic mass(es) that were detected for the first time during the first trimester of pregnancy and were unknown in the preconception period. Preoperative planning included an accurate ultrasound study to assess the number, site, dimensions and characteristics of large mass(es); the myometrial thickness between the mass and the uterine cavity; the evaluation of risks and benefits; a multidisciplinary board with experienced surgeons and patient consent.

The timing of interventions reported in previous studies ranged between the first and second trimester of pregnancy. In our cases, we decided to perform myomectomy during the second trimester, when the risks of spontaneous abortion and preterm contractions are lower. This approach was made possible by the fact that none of our cases needed an urgent procedure, making it the optimal time for non-urgent surgical treatment. Moreover, existing data on anesthetic agents have not been associated with teratogenic effects in humans when standard concentrations are used at any gestational age [17]. 

Regarding the surgical outcomes, in line with the data in the literature, no major postoperative complications occurred and no hysterectomy was needed. Regarding the obstetric outcomes, spontaneous abortion occurred with spontaneous fetal expulsion in only one case, followed by curettage, on the same day of the myomectomy.

The abovementioned case, characterized by fetal demise, differed from the others because of 1. hemorrhagic episodes during the first trimester; 2. the presence of more than 10 myomas and 3. conspicuous intraoperative blood loss leading to anemia, requiring transfusions.

Although this is a single case, such an event suggests that preoperative counseling probably needs to be focused on the risk of abortion and/or hysterectomy if the number of myomas exceeds 10 and/or if the pregnancy is at risk of an abortion due to a hemorrhage in the first trimester. 

Indeed, according to a recent review [18], myomectomy in pregnancy can have a success rate as high as 90%, while the risk of miscarriage or fetal demise after myomectomy is reportedly about 6–7% (13 cases/198 cases). The procedure was successful in eight out of nine of our cases (88.8%), which is consistent with the reported data. 

Intraoperative ultrasounds are useful for assistance during myomectomy in pregnant women [19].

## 5. Conclusions

Our experience confirms that large uterine mass removal is a feasible option during pregnancy when the benefits exceed the risks. Surgical treatment should not be viewed as standard practice, but rather taken into consideration in case of large mass(es), especially those detected for the first time during pregnancy and/or when there is a need to obtain a pathological characterization to exclude the possibility of malignancy. Data from our experience indicated, after surgical treatment, the benign nature of the mass(es), despite their large size being undetected in the preconception period. Unless urgent, surgery should be performed during the second trimester of pregnancy by an experienced team of surgeons. Prenatal counseling should be provided during the first trimester and prior to performing any surgical treatment; the risks and benefits of a surgical procedure during pregnancy should be carefully evaluated and discussed with patients.

## Figures and Tables

**Table 1:** Characteristics of patients.

Case	Age	Ethnicity	BMI	Associated Pathologies	Education
**1**	38	Caucasian	26	Thrombophilia deficit ATIII	High school diploma Housewife
**2**	35	Caucasian	22	Gestational diabetes	High school diploma Housewife
**3**	40	Caucasian	24	Gestational hypertension	High school diploma Housewife
**4**	28	Caucasian	27	None	High school diploma Housewife
**5**	36	Caucasian	27	None	High school diploma Housewife
**6**	38	Caucasian	28	Endometriosis	High school diploma
**7**	38	Black African	24	None	Low education level
**8**	33	Caucasian	30	CMV in pregnancy	High school diploma
**9**	37	Caucasian	34	Gestational hypothyroidism	High school diploma

**Table 2:** Preoperative, intraoperative and postoperative characteristics of masses (SS: subserous; IM: intramural; EBL: estimated blood loss).

Case	Masses > 50 mm	US Characteristics	Intraoperative Data	Histologic Results
**1**	1	90 mm left lateral mass, implantation base of 290 mm. Myometrial thickness: 10 mm Hypervascularity: none	LPT 280 mm SS fundic mass	LEIOMYOMA OF 290 mm
**2**	2	173 × 87 × 116 mm mass, implantation base of 54 mm; 60 × 44 × 56 mm mass, implantation base of 46 mm. Both masses SS right fundic-posterolateral. Myometrial thickness: 11 mm Hypervascularity: none	LPT 200 mm SS fundic mass, with implantation base of 50 mm; 50 mm SS fundic mass; 2 anterior centimetric mass; EBL < 200 cc	LEIOMYOMAS of 4 myomatous nodules, the largest of 190 mm
**3**	2	47 mm × 59 mm isthmic IM mass (dislocating uterine cervix); 21 mm × 26 mm right posterolateral IM mass; 196 × 105 mm right fundic-lateral SS sessile mass, implantation base of 62 mm. Myometrial thickness: 14 mm Hypervascularity: none	LPT 200 mm SS fundic-posterior mass; 2 SS mass of 50–60 mm; 3 SS mass of 20–30 mm; EBL 250 cc	LEIOMYOMAS of 6 myomatous nodules, dimensions range between 10 and 190 mm
**4**	1	220 × 179 × 145 mm fundic right posterolateral SS mass Myometrial thickness: 10 mm Hypervascularity: none	LPT 250 mm SS mass; EBL 450 cc	LEIOMYOMA of 170 mm
**5**	1	160 × 150 × 100 mm left anterolateral SS mass, implantation base of 92 mm. Myometrial thickness: 12 mm Hypervascularity: none	LPT 160 mm mass, implantation base of 80–90 mm; EBL 350 cc	APOPLECTIC LEIOMYOMA of 160 mm
**6**	1	121 × 81 × 73 mm right anterior mass. Myometrial thickness: 13 mm Hypervascularity: none	LPT 120 mm antero-isthmic mass; 30 mm SS anterior mass; EBL 250 cc	LEIOMYOMAS: 1 of 140 mm; 1 of 43 mm
**7**	2	87 × 66 × 49 mm left antero-isthmic IM-SS mass; 146 × 110 × 142 mm right fundic-lateral, implantation base of 89 mm. Myometrial thickness: 10 mm Hypervascularity: none	LPT 150 mm right fundic mass, with a wide implantation base that touches the gestational chamber; 70 mm left antero-isthmic IM mass; other small (10 mm) pedunculated mass	LEIOMYOMAS: 2 intramural nodules of 80–170 mm; Other fragments of 80 mm
**8**	2	123 × 90 × 78.5 mm fundic mass; 78.5 × 64 × 76 mm fundic mass, implantation base of 120 mm. Myometrial thickness: 11 mm Hypervascularity: none	LPT 150 mm right fundic IM-SS mass; 90 mm anterior mass	LEIOMYOMAS: 2 myomatous nodules of 195 mm e 93 mm; 1 fibroid fragment of 155 mm;
**9**	4	157 × 90 mm right fundic-lateral mass; 77 × 76 mm left lateral mass; implantation base of 157 mm; Other small masses observed. Myometrial thickness: 10 mm Hypervascularity: none	LPT 140 mm mass; 120 mm mass; 80 mm mass; 55 mm mass; 20 mm mass; 40 mm mass	LEIOMYOMAS: 140 mm; 120 mm; 80 mm; 55 mm; 20 mm; ADENOMYOMA 40 mm
